# The neuroscience gateway portal: high performance computing made easy

**DOI:** 10.1186/1471-2202-15-S1-P101

**Published:** 2014-07-21

**Authors:** Ted Carnevale, Amit Majumdar, Subha Sivagnanam, Kenneth Yoshimoto, Vadim Astakhov, Anita Bandrowski, Maryann Martone

**Affiliations:** 1 Neurobiology Department, Yale University Medical School, New Haven, CT 06520, USA; 2 San Diego Supercomputer Center, UC San Diego, La Jolla, CA 92093, USA; 3 Center for Research in Biological Systems, UC San Diego, La Jolla, CA 92093, USA; 4 Neuroscience Department, UC San Diego, La Jolla, CA 92093, USA

## 

We present the Neuroscience Gateway (NSG) Portal http://www.nsgportal.org/, which we are developing so that neuroscientists can easily use High Performance Computing (HPC) resources for computationally intensive modeling tasks such as simulating large-scale networks or exploring high-dimensional parameter spaces. Potential users of HPC resources typically face a high entry barrier because of numerous complex side-issues, such as applying for CPU time, installing and configuring simulation software, remote authentication, data transfer and retrieval, batch system management, and administrative policies--all of which may vary from one site to another. The NSG Portal reduces this entry barrier by streamlining administrative procedures and providing a convenient web browser-based interface that hides technical details, and thereby enables investigators to focus on scientific issues relevant to their research.

Brian, NEST, NEURON, PGENESIS, and PyNN are now installed and available through the Portal; suggestions for other tools are welcome. NSG's entry point is a simple browser-based interface through which users can upload input files or model source code, specify code-specific input files, specify job submission parameters (such as number of cores and nodes, expected wall clock time for job completion), monitor the status of submitted jobs, and extract and download output files. Files may be uploaded as plain text, and collections of files in a pkzip archive. Job results are downloadable as a pkzip archive of that job's working directory, including input and output files. Users are notified of job completion by email.

We have been obtaining time allocations for the NSG Portal on HPC resources from XSEDE, an NSF-supported partnership of academic institutions whose goal is to facilitate sharing of computing resources, data, and expertise. Through XSEDE's national-level peer review process, our application for HPC time was awarded 1.6 million "service units" (~ 1.6 million CPU hours) for the current year. From this, each NSG user is given an initial allotment of 5000 core hours per year, subject to adjustment in light of actual usage by all NSG users. Those who have independently obtained their own HPC allocations may manage that time via the NSG.
